# Standards and Impact of Hematopathology in Myelodysplastic Syndromes (MDS)

**DOI:** 10.18632/oncotarget.185

**Published:** 2010-11-16

**Authors:** Peter Valent, Attilio Orazi, Guntram Büsche, Annette Schmitt-Gräff, Tracy I. George, Karl Sotlar, Berthold Streubel, Christine Beham-Schmid, Sabine Cerny-Reiterer, Otto Krieger, Arjan van de Loosdrecht, Wolfgang Kern, Kiyoyuki Ogata, Friedrich Wimazal, Judit Csomor, Judit Várkonyi, Wolfgang R. Sperr, Martin Werner, Hans Kreipe, Horny Hans-Peter

**Affiliations:** ^1^ Department of Internal Medicine I, Division of Hematology & Hemostaseology, Medical University of Vienna, Austria; ^2^ Ludwig Boltzmann Cluster Oncology, Vienna, Austria; ^3^ New York Presbyterian Hospital, Weill Cornell Medical Center, New York, NY, USA; ^4^ Institute of Pathology, Hannover Medical School, Hannover, Germany; ^5^ Department of Pathology, University of Freiburg, Germany; ^6^ Department of Pathology, Stanford University School of Medicine, Stanford, CA, USA; ^7^ Institute of Pathology, University of Munich; ^8^ Institute of Pathology, Medical University of Vienna; ^9^ Institute of Pathology, Medical University of Graz, Austria; ^10^ First Department of Internal Medicine, Elisabethinen Hospital Linz, Austria; ^11^ VU University Medical Center, Amsterdam, The Netherlands; ^12^ MLL Munich Leukemia Laboratory, Munich, Germany; ^13^ Division of Hematology, Department of Medicine, Nippon Medical School, Tokyo, Japan; ^14^ Department of Obstetrics and Gynaecology, Medical University of Vienna; ^15^ Institute of Pathology, Semmelweis University, Budapest, Hungary; ^16^ Department of Hematology, Semmelweis University, Budapest, Hungary; ^17^ Institute of Pathology, Ansbach, Germany

**Keywords:** MDS, hematopathology, diagnostic criteria, prognostication

## Abstract

The diagnosis, classification, and prognostication of patients with myelodysplastic syndromes (MDS) are usually based on clinical parameters, analysis of peripheral blood and bone marrow smears, and cytogenetic determinants. However, a thorough histologic and immunohistochemical examination of the bone marrow is often required for a final diagnosis and exact classification in these patients. Notably, histology and immunohistology may reveal dysplasia in megakaryocytes or other bone marrow lineages and/or the presence of clusters of CD34-positive precursor cells. In other cases, histology may reveal an unrelated or co-existing hematopoietic neoplasm, or may support the conclusion the patient is suffering from acute myeloid leukemia rather than MDS. Moreover, histologic investigations and immunohistology may reveal an increase in tryptase-positive cells, a coexisting systemic mastocytosis, or bone marrow fibrosis, which is of prognostic significance. To discuss diagnostic algorithms, terminologies, parameters, and specific issues in the hematopathologic evaluation of MDS, a Working Conference involving a consortium of US and EU experts, was organized in June 2010. The outcomes of the conference and resulting recommendations provided by the faculty, are reported in this article. These guidelines should assist in the diagnosis, classification, and prognostication in MDS in daily practice as well as in clinical trials.

## INTRODUCTION

Myelodysplastic syndromes (MDS), also known as myelodysplastic neoplasms, are clonal disorders characterized by a maturation defect in myelopoietic progenitor cells, peripheral cytopenia(s), and clonal instability with an enhanced risk of transformation to secondary acute myeloid leukemia (sAML) [1-3]. MDS are classified according to criteria first provided by the French-American-British (FAB) working group, and later integrated into the World Health Organization (WHO) proposal [4-6]. The WHO classification of MDS was updated in 2008 and provides robust criteria for the discrimination of MDS variants from each other [6]. In addition, minimal diagnostic criteria for MDS have been proposed [6,7]. These criteria allow discrimination of MDS from all other neoplastic or reactive disorders that can also produce cytopenia(s) or/and dysplasia. Diagnostic prerequisite-criteria for MDS include a) persistent significant cytopenia(s), b) cytologic or cytogenetic evidence of myelodysplasia, and c) exclusion of all other conditions and disorders producing cytopenia(s) and/or dysplasia [7].

From a practical point of view, the diagnosis MDS is established in a step-wise procedure (Table 1). In a first step, minimal diagnostic criteria need to be fulfilled. Then, the subtype of MDS according to WHO criteria should be defined. Next, the patient is examined for individual risk factors and scores, in order to establish the overall risk profile, preferably by the international prognostic scoring system (IPSS) [8] and WHO-prognostic scoring system (WPSS) [9]. Finally, if applicable, treatment-scores are applied to define the optimal therapeutic approach (Table 1).

**Table 1:** Step-wise approach in the diagnosis, prognostication, and treatment in MDS

1. Minimal Diagnostic Criteria	Establish the Diagnosis MDS
2. FAB and WHO Classification	Establish the MDS Subtype/Variant
3. IPSS, WPSS and other Scores	Establish the Risk of Transformation (AML)
4. Patient-related Risk Factors*	Estimate (AML-free) Survival Times
5. Therapy-related Scores	Establish the Treatment Plan
6. Response Criteria	Determine Treatment Responses
MDS, myelodysplastic syndromes; FAB, French-American-British working group; WHO, world health organization; IPSS, international prognostic scoring system; WPSS, WHO-adapted prognostic scoring system. *Patient-related risk factors include variables not covered by the IPSS/WPSS, such as age or comorbidity.	

In most patients with MDS, the bone marrow (BM) smear reveals marked dysplasia in one or more major hematopoietic lineage/s (erythroid, granulocytic, mega-karyocytic)[10,11]. With regard to megakaryopoiesis, a frequent problem in MDS is that megakaryocyte numbers in BM smears may be rather low. Monocytosis or/and an increase in blast cells may also be detected in BM smears [10,11]. Typical blood count abnormalities recorded in MDS include macrocytic anemia, bi- or pancytopenia, and signs of dysplasia such as abnormal hypogranulated or/and hypolobated neutrophils (e.g. Pseudo-Pelger-Huet cells). In most patients, a provisional diagnosis of MDS can be established on examination of blood and BM smears. In other patients, no prominent dysplasia is found but an abnormal karyotype is detected, leading to the conclusion the patient may suffer from MDS [7]. However, there are also patients with normal karyotype in whom it is difficult to define whether cytopenia or dysplasia would indeed result from an underlying MDS, a prephase of MDS, or from another hematologic or even non-hematologic disease [7]. In other patients, it is difficult to discriminate between advanced MDS and AML, or a myelodysplastic/myeloproliferative overlap disease (MDS/MPN).

In all these instances, histological and immunohistochemical examination of BM biopsies, a diagnostic approach which was often underestimated in the past, contributes essentially to the diagnosis, classification, and prognostication of (suspected/provisional) MDS [7,12,13]. In order to discuss current standards in the evaluation of MDS by histology and immunohistochemistry, a Working Conference was organized in June 2010. The participating faculty discussed current and novel diagnostic procedures and markers, related criteria, and diagnostic algorithms. The outcome was formulated into consensus statements. The level of consensus was defined as percent agreement (percent of faculty members agreed). A summary of consensus statements and related recommendations are presented in this article.

## BASIC RECOMMENDATIONS

The faculty agreed 100% that it is essential for the pathologist to receive BM samples together with relevant clinical information and laboratory parameters, a complete blood count (CBC) with differential count as well as unstained BM and peripheral blood (PB) smears, in order to establish the correct diagnosis. The BM biopsy specimen (iliac crest) should be of adequate length (≥ 2 cm). Specimens should be fixed in neutral-buffered formalin, decalcified in edetic acid, and embedded in paraffin-wax [12]. Alternative fixation and embedding techniques are acceptable provided that the quality of the morphologic stain and the section immunoreactivity are both adequate. Standard routine stains include H&E and/or Giemsa, and Gömöri's silver stain for the evaluation of fibrosis [12,14]. Special stain for naphtol AS-D chloroacetate esterase (CAE) is helpful in determining the erythroid:myeloid ratio, and may provide a first hint for the presence of a CAE-negative (lymphoid or blast) infiltrate or/and granulocyte dysplasia. The BM cellularity is reported as percentage of section-area according to the standard proposed by Tuzuner and Bennett [15]. A widely used approach is to classify the estimated BM cellularity as ‘normocellular’, ‘hypocellular’, or ‘hypercellular’. Such estimate should be based on age-adapted normal values proposed for the groups 20-30 years (60-70% cellularity), 40-60 years (40-50%), and ≥ 70 years (30-40%) [16]. In each case, a Prussian blue stain (iron stain) should be performed on a BM smear.

## LINEAGE ASSESSMENT BY IMMUNOHIS TOCHEMISTRY AND RECOMMENDED MARKERS

The application of immunohistochemical (IHC) markers is recommended in all patients with (suspected) MDS [7,12,14]. Because of subclone-formation and phenotypic diversity, it may sometimes be necessary to apply multiple markers for one lineage (cell type) even in the same patient. The participants agreed that all major BM lineages should be examined by immunohistochemistry in (suspected/provisional) MDS. The minimal panel recommended for all patients includes CD34 (progenitor/precursor cells), CD117/KIT (progenitor/precursor cells, mast cells), tryptase (mast cells, immature basophils), one megakaryocyte marker (CD61 or CD42b), CD20 (B-lineage), CD3 (T cells), and glycophorin-A or -C (Table 2). Additional (lineage-specific) markers are applied depending on initial staining results and further clinical and laboratory parameters. Such additional markers are essential when the diagnosis MDS is in question or another co-existing neoplasm is suspected. Sometimes, the application of an antibody against myeloperoxidase (MPO), CD25, CD33, or lysozyme is helpful [12]. Using the minimal marker-panel proposed, the pathologist can also study endothelial cells (CD34+/CD31+) and may report on microvessel density [17].

**Figure 1:**
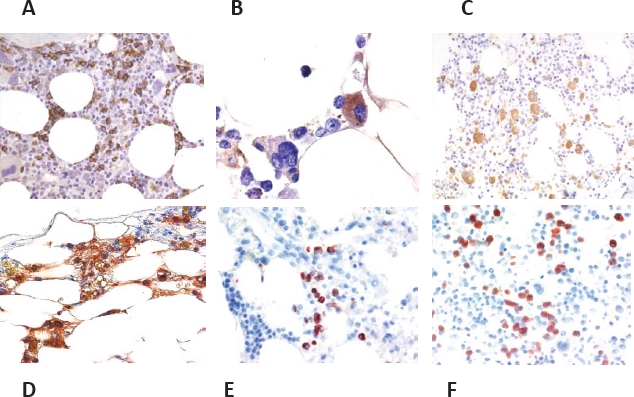
Application of lineage-associated immunohistochemical markers in MDS Bone marrow sections obtained from patients with MDS stained with antibodies against CD34 (A, B), CD42b (C), tryptase (D), 2D7 (E), and eosinophil major basic protein, EMBP (F). The CD34 stain is useful for detection of immature precursor cells (blast cells) in patients with RAEB (A). Megakaryocytes and megakaryoblasts may also stain positive for CD34 in MDS (B). A preferred marker of the megakaryocyte lineage is, among other, CD42b (C). The tryptase stain may reveal an increase in mast cells (D), whereas 2D7 (E) is specific for basophils, and EMBP (F) is useful for the visualization of eosinophils in patients with MDS.

The CD34 stain is useful for the detection of clusters and/or aggregates of immature myeloid cells which should be reported if present [18,19]. In instances where blast cells are CD34-negative cells, KIT is recommended as an alternative (additional) marker [7,12]. However, because KIT is also expressed by a proportion of proerythroblasts [20], evaluation may be difficult in erythroid-rich cases. The faculty agreed that it is essential to report on the estimated percentage of CD34+ cells (percent of all nucleated cells) in each case of (suspected or overt) MDS. The faculty also agreed that any multifocal accumulation (abnormal clustering) of CD34+ cells (Figure 1A) must be regarded abnormal and potentially indicative of an MDS. Without immunostaining, i.e. by morphology alone, it is quite difficult to identify an abnormal localization of immature precursor cells (ALIP) [21], in particular when there is sub-optimal fixation of the trephine. An easier and probably more accurate feature to describe and record in (suspected/provisional) MDS is the ‘Abnormal Multifocal Accumulation (clustering) of CD34+ precursor cells' (AMA-CD34) (Figure 1A), which should thus replace the reporting on ALIP [7,12]. Nevertheless, histologic blast cell recognition remains important because subpopulations or (rarely) the entire population of blasts may be CD34-negative cells. If an increase of blast cells can neither be documented by histomorphology nor in bone marrow smears, the phrasing should change to the more appropriate term of ‘CD34+ progenitor/precursor cells’.

Megakaryocyte-reactive antibodies are useful for the visualization of normal and abnormal megakaryocytes, and their (abnormal) accumulation in the BM [12,14]. Both small-sized megakaryocytes (dwarf forms including micromegakaryocytes) and megakaryoblasts can be identified using this approach (Figure 1B and 1C). Notably, in almost all patients with MDS, megakaryocytes show both atypical cytologic features and abnormal distribution [7,12]. The faculty agreed that CD61 and CD42b can be considered as ‘standard megakaryocyte markers’ in MDS (Table 2). The linker for activation of T cells (LAT), van Willebrand factor (vWF, factor VIII antigen), CD25, and CD31 are also expressed in megakaryocytes. Furthermore, the CD34 antigen may be detectable in (immature) megakaryocytes and megakaryoblasts in MDS (Figure 1B and 1C). However, CD34-expression is not a specific feature of MDS-megakaryocytes. On the other hand, most megakaryocytes in the normal/reactive BM usually are CD34-negative, so that a clear-cut expression of CD34 in a majority of megakaryocytes must be regarded as phenotypic aberrancy supporting the conclusion the patient suffers from a myeloid neoplasm such as MDS.

**Table 2:** Immunohistochemical markers recommended for the evaluation of MDS

Marker CD	Antigen	Cell Type(s)	Recommended by Faculty (Consensus Level %)*	Value/Impact in MDS
CD34	HPCA-1	Progenitor cells, endothelial cells	100%	RAEB-2 vs AML, hypoplastic MDS, microvessel density, megakaryoblasts
CD117	KIT/SCFR	Progenitor cells, mast cells, immature erythroblasts	90%	Mastocytosis, SM-MDS, CD34-negative clones
CD31	PECAM-1	Megakaryocytes endothelial cells	<50%	Abnormal megakaryocytes, dwarf forms Microvessel density
CD42	GPIX	Megakaryocytes	65%	Abnormal megakaryocytes, dwarf forms
CD61	VNRß	Megakaryocytes	90%	Abnormal megakaryocytes, dwarf forms
n.c.	Glycophorin-A/C	Erythroid cells	65%	Erythroid hyperplasia, AML M6 vs MDS
n.c.	Myeloperoxidase	Myeloid cells	70%	Neutrophilic cells, maturation defect
n.c.	Tryptase	Mast cells, immature basophils	85%	Mastocytosis, SM-MDS Basophilia MDS vs MPN
CD14	LPSRr	Monocytes, subset of macrophages	95%	CMML vs MDS
CD68R	PGM1	Macrophages, histiocytes, monocytes, mast cells	55%	CMML vs MDS
CD3	TCR	T cells	85%	T cell neoplasm
CD20	B1	B cells	85%	B cell neoplasms
CD25	IL2Ralpha	Megakaryocytes, histiocytes, T cells, atypical mast cells	85%	Abnormal megakaryocytes SM-MDS
2D7	2D7-antigen	Basophils	90%	Basophilia, basophilic leukemia

The tryptase stain is useful to detect loosely scattered mast cells which are increased in a majority of patients with MDS and may show a spindle-shape morphology (Figure 1D) [12]. Serum tryptase levels are also elevated in a group of patients with MDS [22]. If mast cells form compact clusters in the BM and/or express CD25, or the serum tryptase level is markedly elevated, it is appropriate to perform *KIT* mutation analysis [7,12]. In such cases, a coexisting (occult) systemic mastocytosis is detected quite frequently.

So far, only very few immunohistochemical markers sufficient for the evaluation of basophils and eosinophils in BM sections are available. For basophil evaluation and counting, 2D7 and BB1 (basogranulin) are recommended antigens (Figure 1E) [23,24] while eosinophils can be visualized using an antibody against eosinophil major basic protein (EMBP) (Figure 1F). Whereas a slight or moderate increase of eosinophils is often seen in reactive and neoplastic disease states, constant basophilia is uncommon in reactive states and thus regarded as a potential indicator for the presence of a myeloid neoplasm. However, no robust studies employing basophil or eosinophil IHC markers in MDS have been conducted so far. The faculty agreed that such investigations should be performed in order to examine the utility of such markers and the impact of BM eosinophilia and basophilia in MDS. If BM or blood eosinophilia is substantial in MDS, the BM should be examined for the presence of rearrangements involving *PDGFR* and *FGFR* genes.

## IMPACT OF HISTOPATHOLOGIC PARAMETERS IN THE DIAGNOSIS OF MDS

The diagnosis of MDS is primarily based on the presence of persistent (of at least 6 months duration) cytopenia(s), cytomorphologic dysplasia in one or more major BM lineages (erythroid, granulocytic, megakaryocytic), and exclusion of other potential disorders that can produce cytopenia and dysplasia [7]. To address these criteria and thus establish the exact diagnosis, it is essential to examine a representative BM biopsy section by histology and immunohistochemistry. First, the BM histology may reveal a myeloid neoplasm other than MDS, or MDS with a coexisting neoplasm (hematopoietic or non hematopoietic). Likewise, in patients with provisional RAEB-2, the BM biopsy may reveal a final diagnosis of AML (e.g. by demonstrating sheets of CD34+ cells). In other cases of (provisional) MDS, a co-existing systemic mastocytosis (SM) will be detected, leading to the final diagnosis of SM-MDS [12]. Another example is the discrimination between aplastic anemia, hypoplastic MDS, and hypoplastic AML [3]. Again, the final diagnosis in these patients cannot be established without a thorough investigation of BM sections. Finally, the BM histology may reveal a myeloproliferative neoplasm or an MDS/MPN overlap disease, which can be accompanied by the *JAK2* mutation V617F [25].

After having excluded other (differential) diagnoses in a cytopenic patient, the pathologist will examine the BM for signs of dysplasia in detail. Whereas dysplasia of erythroid cells and neutrophils is examined preferentially in BM and PB smears, megakaryocyte dysplasia can often be assessed more accurately in BM sections [3,7,12]. This is often essential, especially when BM smears contain only a few megakaryocytes. The faculty agreed that dysplasia should count as an MDS-specific criterion, when ≥ 10% of cells in a given lineage show clear signs of dysplasia, as has been proposed by the WHO and other working groups [5,7]. However, as mentioned above signs of dysplasia in one or even more lineages may also be recorded in a variety of other hematopoietic and even non-neoplastic conditions, such as vitamine B12 or folate deficiency, viral infections, or chronic inflammation.

## IMPACT OF HISTOPATHOLOGY IN THE CLASSIFICATION OF MDS

### a. Evaluation of megakaryopoiesis and megakaryocyte dysplasia

As mentioned above it may be essential to confirm or reveal megakaryocyte dysplasia in the BM histology. Thus, the diagnosis of multi-lineage dysplasia, a major diagnostic determinant in the WHO classification [5] often depends on a thorough assessment of megakaryocytes in BM sections because BM smears often contain only low numbers of megakaryocytes in MDS. The presence of dwarf megakaryocyte forms (including micromegakaryocytes) and abnormalities in their distribution as frequently seen in MDS can be best established by examining BM sections immunostained with one or more megakaryocyte marker(s) such as CD61, CD42b, or CD31 (Figure 1C) [12,14]. As previously mentioned, aberrant expression of CD34 in megakaryocytes can be seen in MDS (Figure 1D). However, this phenomenon is neither specific for MDS nor related to a specific subtype of MDS. Small megakaryocytes with markedly hypolobated nuclei (‘mononuclear megakaryocytes’) are typically found in patients with the 5q- anomaly. However, there is no absolute correlation between a particular megakaryocyte-morphology and a certain cytogenetic abnormality in MDS.

### b. Evaluation of CD34+ cells and delineation between low risk MDS and high risk MDS

An important diagnostic approach in MDS is the evaluation of CD34+ progenitor/precursor cells in BM histologic sections. This approach is helpful (often essential) for the delineation between low risk MDS (RA, RARS, RCMD) and high risk MDS (RAEB-1, RAEB-2) [18,19]. In each case, the estimated percentage of CD34+ cells (called blast cells when BM smears confirm blast cell morphology) should be reported. Abnormal multifocal accumulation of CD34+ cells (AMA-CD34) is only seen in patients with high risk MDS [3,7,12]. In case of CD34-negative progenitor cells (blasts), KIT/CD117 can also be employed as alternative marker antigen. However, KIT is also expressed on other BM cells including mast cells and a subset of (immature) erythroblasts [20].

### c. Hypoplastic MDS (MDS-hypo)

**Figure 2:**
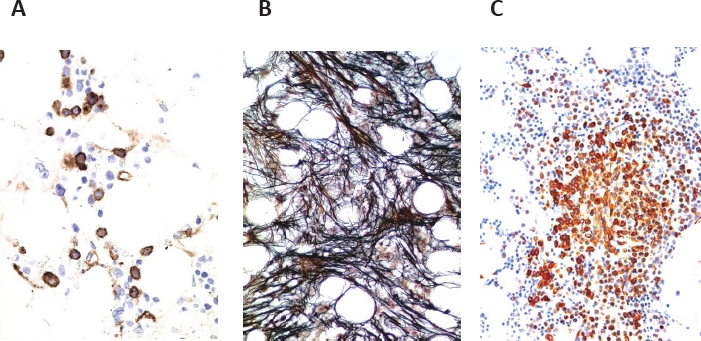
Diagnostic potential of histomorphological features in MDS and delineation of MDS subtypes A: Immunohistochemistry reveals an increase in CD34+ precursor cells in a patient with hypoplastic MDS. Note focal clustering of CD34+ precursor (blast) cells in the bone marrow section. B: The fibrotic form of MDS (MDS-F) as evidenced by Gömöri's silver stain (grade III). C: MDS associated with systemic mastcytosis (SM-MDS) as evidenced by staining the bone marrow section for mast cell tryptase. Note the compact infiltrate of spindle shaped tryptase-positive mast cells in this patient.

Another proposed subtype of MDS that can only be diagnosed by histology is hypoplastic MDS [3,12]. The faculty agreed that this subtype should be recognized as a separate variant of MDS and should be defined by robust criteria. In fact, MDS should be called hypoplastic MDS when a) minimal diagnostic criteria for MDS [7] are fulfilled, b) the BM section is hypocellular compared to age-matched normal BM cellularity [16], and c) causes and therapies producing transient cytopenia have been excluded. Cases of therapy-related MDS can also present with hypocellular marrows. Sometimes, it may be difficult to differentiate between hypoplastic MDS and hypoplastic AML [3,13]. Histologic or immunohistochemical identification of immature precursor/blast cells (by antibodies against CD34 and KIT) is essential in identifying these cases, and to established the final diagnosis of hypoplastic MDS or hypoplastic AML (Figure 2A) [12,26].

### d. MDS with bone marrow fibrosis (MDS-F)

Another prognostic variable in MDS is BM fibrosis [27-29]. The degree of fibrosis should be assessed according to the European Consensus Grading System also known as EURO-Score[16] and should be reported in all MDS patients. The presence of BM fibrosis EURO-Score grade II or grade III is required to call the disease MDS-F (Figure 2B). The faculty then discussed whether MDS patients presenting with such marked BM fibrosis should be regarded as a separate category of MDS. However, after intensive discussion, the faculty agreed by 80% that the term MDS-F would be appropriate, but MDS-F should not be regarded as a separate variant of MDS. Rather, in each MDS variant and subvariant, the final diagnosis should include the appendix ‘-F’ when fibrosis of ‘grade II’ or ‘grade III’ was recorded (e.g. RCMD-F, RAEB-F) (Figure 2B). The faculty also agreed that the presence of fibrosis (-F) in MDS should be included in the final pathology report in each case because of its well known prognostic significance [27-29]. Moreover, in these patients, the clinician and the pathologist have to look for (additional) signs of myeloproliferation, i.e. increased spleen size, leukocytosis with left shift, marked eosinophilia and/or basophilia, marked thrombocytosis, histologic features of an MPN, *JAK2* V617F, or/and a markedly elevated serum tryptase level (>100 ng/ml) [7,12,25,30]. When two or more of these additional features are recorded, the diagnosis of MDS is in question, and the more likely final diagnosis may be an MDS/MPN overlap disease, an associated/pre-existing MPN, mast cell disease (mastocytosis), or even an unusual MPN with BM dysplasia (Table 3). In other words, MDS-F has to be delineated from various differential diagnoses including primary myelofibrosis (PMF). The faculty agreed that the term ‘myelofibrosis’ should be avoided in patients with MDS-F.

**Table 3:** Impact of Myeloproliferative Features recorded in MDS & Differential Diagnoses

Feature in Overlap-Clusters A-D*	Examples of Differential Diagnoses**
A: MEGA/FIBRO-Cluster Bone Marrow (BM) Fibrosis II or III*** Thrombocytosis >600,000/μL Palpable Splenomegaly Abnormal clustering of megakaryocytes Markedly elevated CFU-GM *JAK2 V617F*	a) 5q- syndrome or RARS-T b) MDS/MPN Overlap Disease c) MPN/PMF with BM dysplasia
B: EO/BASO-Cluster Leukocytosis with Neutrophilia Marked Left Shift in BM or blood smears Marked Eosinophilia and/or Basophilia *PDGFRA/B* mutant *FGFR* mutant	a) MDS-U, MDS-eo b) MDS/MPN Overlap Disease c) Eosinophilic Leukemia or Basophilic Leukemia with BM dysplasia
C: MAST CELL-Cluster Atypical CD25+ Mast Cells Markedly increased Serum Tryptase Urticaria Pigmentosa *KIT* D816V	a) MDS with increase in mast cells b) Myelomastocytic Overlap Disease or SM-MDS d) SM with BM dysplasia, SSM
D: MONO-Cluster BM or blood Monocytosis (>1,000/μL) Marked increase in CD14+ cells Autonomous CFU-GM growth	a) MDS with mild monocytosis b) CMML (Overlap) c) Monoblastic Leukemia (AML)
*Features are clustered according to potential differential diagnoses and involvement of certain hematopoietic lineages. When 2 or more of the features from one cluster are detected, the diagnosis MDS is in question and the more likely diagnosis is an overlap or another unrelated malignancy. **Each 3 examples of a typical differential diagnosis are depicted: a) MDS-type disease, b) MDS/X overlap, and c) another unrelated disease = without coexisting frank MDS. ***Fibrosis should be graded according to the Euro-Score.^15^ MDS, myelodysplastic syndromes; RARS-T, refractory anemia with ring sideroblasts and thrombocytosis; MDS/MPN, myeldysplastic/myeloproliferative overlap disorder; CFU-GM, granulocyte/macrophage colony-forming unit; MDS-U, unclassifiable MDS; MDS-eo, MDS with marked eosinophilia; PDGFR, platelet derived growth factor; SM, systemic mastocytosis; CMML, chronic myelomonocytic leukemia; AML, acute myeloid leukemia, SSM, smouldering SM.	

### e. MDS associated with systemic mastocytosis (SM-MDS)

In a very small group of patients with MDS, a co-existing SM is detected (Figure 2C) [12,31-33]. In most of these patients, an increased serum tryptase level (>20 ng/ml) and the *KIT* mutation D816V are found, and several of them present with typical skin lesions of the urticaria pigmentosa type. If this is not the case, isolated bone marrow mastocytosis (BMM) may be diagnosed [34,35]. The detection of *KIT* D816V is helpful in these cases, as the mutant is rarely if ever detectable in MDS cases without SM. It is noteworthy that *KIT* mutation analysis can not only be performed in BM aspirates but also by using paraffin-embedded material.

The diagnosis SM-MDS is a provisional one because both the SM component and MDS components of the disease need to be subclassified according to WHO criteria [34,35]. An important aspect is that patients with advanced SM including smouldering SM (SSM) and aggressive SM, often present with cytopenia and mild dysplasia [30,34,35]. Therefore, it is of importance to confirm the diagnosis MDS in these patients by using robust histologic, cytomorphologic, and cytogenetic parameters. Relatively strong reliable indications for an MDS include a macrocytic transfusion-dependent anemia, an increase in myeloblasts (CD34+ cells), and/or the presence of an MDS-related cytogenetic abnormality. The prognosis in patients with SM-MDS is variable. In those in whom BMM is diagnosed (BMM-MDS), the prognosis is usually defined by the MDS component of the disease [33]. However, in patients with ASM-MDS or MCL-MDS, the mast cell disease subclone may be the prognostically (more) important disease-component [31-33].

### f. Erythroid-predominant MDS (MDS-Ery)

In a small group of patients with advanced MDS, erythropoiesis is clearly predominant, so that the question arises whether the patient is suffering from MDS or from erythroleukemia (formerly termed AML-M6) [36]. The classical definition of erythroleukemia relates to the percentage of blast cells in the non-erythroid compartment of the BM (≥ 20% by WHO criteria) [37-39]. The faculty discussed these criteria, and specifically asked whether the percentage of blasts among all nucleated BM cells should also count as criterion of AML M6 in these patients. The faculty agreed that it may be appropriate to report on marked erythroid hyperplasia (>50% erythroid cells in the BM) in all cases, and to regard cases with such erythroid predominance as a provisional subcategory of MDS (proposed: erythroid-predominant MDS = MDS-Ery) when criteria for AML are not (yet) fulfilled. The faculty also agreed that the traditional definition of erythroleukemia (M6) should still be recommended as a global standard, but that it may be appropriate to start a discussion to modify this definition in the future, and to regard only those cases as frank AML M6 in whom blast cells account for ≥ 20% of all nucleated BM cells (instead of ≥ 20% of all non-erythroid cells). All other cases with erythroid predominance but <20% blasts (of all nucleated BM cells) would then be called MDS-Ery (e.g. RAEB-1-Ery). In cases of MDS-Ery, the blast cell percentage obtained from counting non-erythroid marrow cells, represents a prognostically significant variable [36].

## THE OVERLAP DISORDERS

Three overlap conditions were discussed in detail by the faculty. Each of these disorders is characterized by massive expansion of more or less dysplastic cells in a distinct hematopoietic cell lineage.

### a. Chronic Myelomonocytic Leukemia (CMML)

Traditionally, CMML is defined by absolute monocytosis (>1000/μL), myeloid dysplasia, and exclusion of AML [25,40-43]. The faculty agreed that an absolute monocytosis is a robust criterion of CMML. However, in patients with high leukocyte counts, the percentage of monocytes may be a more reliable (robust) parameter. Therefore, the faculty recommends that in forthcoming classification proposals, the relative monocyte count (e.g. >10%) should be considered as an additional criterion, especially when leukocyte counts are very high. The WHO classification discriminates CMML-1 (less than 10% blasts in BM) from CMML-2 (10-19% blasts). The faculty agreed that a thorough histomorphological and immunohistochemical (or flow cytometric) investigation of BM cells is essential for the diagnosis of CMML (recommended marker: CD14)[43] and delineation between CMML-1 and CMML-2 (recommended marker: CD34), which may be difficult as monoblasts or promonocytes often lack CD34 [41,42]. Because of their frequent CD34-negativity and the difficult histologic identification of promonocytes, the separation of CMML-2 from acute myelomonocytic or acute monocytic leukemia may sometimes be difficult, particularly in the absence of flow cytometry and/or cytogenetics. Notably, in these patients, a thorough examination of a good quality BM smear as well as flow cytometry may be helpful to reach the final diagnosis.

A detailed cytogenetic and molecular analysis is required in all patients with CMML. The panel of markers to be applied in these patients depends on involvement of additional lineages (apart from monocytes): for example, in those with marked (PB or BM) eosinophilia, neoplastic cells should be screened for the presence of *PDGFR*- or *FGFR* rearrangements. When mast cells are reported to be abnormal and/or increased in number, PB and BM cells should be examined for the presence of *KIT* D816V. In mutant-positive cases, the tryptase-stain usually reveals (an otherwise overlooked or occult) co-existing BM mastocytosis (BMM). In other patients, mutations in *RAS, JAK2, TET2, CBL, or RUNX1* are found [44,45]. Sometimes, the monocyte compartment expands or even shrinks (spontaneously) over time, so that the diagnosis may change (e.g. from RA to CMML). An increase in CD34+ cells is usually followed by evolution to AML.

### b. Myelomastocytic Leukemia (MML)

This overlap disease is an extremely rare condition defined by an advanced myeloid neoplasm and prominent secondary expansion of clonal mast cells [31,46-48]. Major differential diagnoses are (primary) mast cell leukemia (MCL), mastocytosis with associated MDS (SM-MDS), and basophilic leukemia [31,48]. MML is defined by a) an advanced MDS (or another advanced myeloid neoplasm), b) at least 10% atypical mast cells in BM or PB smears, and c) exclusion of a primary mast cell disease, i.e. SM criteria are not fulfilled in these cases [31,46]. In contrast to MCL and SM-MDS, no focal mast cell aggregates are present; only a diffuse infiltration of the BM by atypical immature mast cells is found in MML [31,46]. In addition, no *KIT* mutation at codon 816 is found in MML, and neoplastic mast cells usually are CD25-negative cells.^46^ MML can also be discriminated from basophilic leukemia by immuno-phenotyping, as mast cells are KIT+/CD34-/2D7- cells, whereas basophils are KIT-/CD34-/2D7+ cells.

#### c. MDS/MPN overlap disorders with basophilia and/or eosinophilia

Demonstration of prominent basophilia and/or eosinophilia in the PB and/or the BM in an MDS patient is an important diagnostic checkpoint [49-51]. In these patients it is appropriate to exclude or reveal certain myeloid neoplasms defined by distinct cytogenetic and/or molecular markers. In rare cases, an accelerated phase of Ph+ CML, advanced mast cell neoplasm, or 8p11 syndrome involving the *FGFR* are detectable [48-50]. More frequently, myeloid neoplasms with abnormalities in the *PDGFRA*- or *PDGFRB* genes, are detected[49-51], which is of clinical importance, as most of these patients respond to imatinib [52,53]. In some of these cases, BM dysplasia is found, so that it is justified to diagnose an MDS/MPN overlap disease. In the majority of cases, however, no prominent BM dysplasia is recorded.

### IMPACT OF HISTOPATHOLOGY IN THE PROGNOSTICATION OF MDS

There are a number of important prognostic parameters that should be addressed and reported by the hematopathologist when evaluating BM sections in MDS patients. Most important prognostic histopathologic variables in MDS are the presence of AMA-CD34 (increase in CD34+ cells), marked BM fibrosis, and an overt MDS/MPD overlap disease, including myelomastocytic transformation.

### ABERRANT PHENOTYPES IN MDS: ROLE OF BM IMMUNOHISTOCHEMISTRY, NOVEL TECHNOLOGIES, AND COMPARISON to FLOW CYTOMETRY

**Figure 3:**
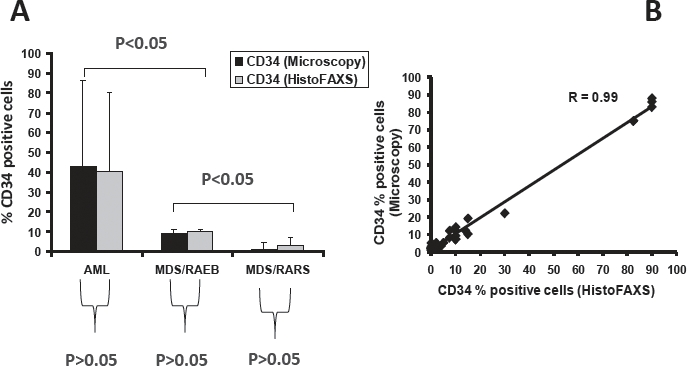
Evaluation of CD34+ cells in the MDS marrow by Tissue-FAXS A. Percentage of CD34+ cells in the bone marrow of patients with acute myeloid leukemia (AML), MDS subtype RAEB, and MDS subtype RARS. The percentage of CD34+ cells was determined (estimated) in CD34-stained bone marrow sections by microscopy (black bars) as well as by TissueFAXS using HistoFAXS software (grey bars) in blinded fashion. As visible, there was an excellent correlation when comparing the two techniques of quantification of CD34+ progenitor cells. B:.Correlation of CD34+ cell counts in all patients (correlation coefficient R=0.99).

Compared to flow cytometry, only very little is known about aberrant antigenic expression by immunohistology in MDS. As mentioned before, expression of CD34 in megakaryocytes can occur in MDS, but is not a specific feature. Compared to normal (mature) blood basophils, BM basophils in MDS often are immature cells and may therefore synthesize and express substantial amounts of tryptase [54]. Aberrant expression of CD25 in BM mast cells has also been described. However, none of these aberrant immunohistochemical features are specific for MDS. Further examples are a loss of CD34 in myeloblasts or loss of MPO in more mature myeloid cells in the BM in MDS. Again, these features are not specific for MDS. The faculty agreed that it may be important to confirm aberrant markers discovered by flow cytometry [55] in MDS patients by an immunohistochemistry approach in BM sections in these patients. However, in contrast to flow cytometry, immunohistochemical parameters cannot usually be assessed by computerized multi-color-staining. One such future technique that may be helpful to overcome this problem may be tissue FAXS [56]. The faculty agreed that novel technologies to better quantify immunoreactivity and to detect aberrant immunophenotypes of BM lineages in MDS would be beneficial and that this should be further investigated in preclinical research programs. In one such pilot project presented by a member of the faculty, tissue FAXS allowed an accurate assessment of the numbers of CD34+ BM cells (Figure 3). There are a number of markers that are expressed aberrantly and should be examined in BM cells in MDS in future projects. Most interesting markers to be examined in CD34+ blast cells may be HLA-DR, CD7, and CD45 [55]. In fact, these markers clearly show aberrant expression in CD34+ blast cells in flow cytometry studies.

### IDIOPATHIC CYTOPENIA (ICUS) AND IDIOPATHIC DYSPLASIA (IDUS) OF UNDETERMINED SIGNIFICANCE

A diagnostic challenge are patients who do not fulfil minimal diagnostic criteria for MDS but are suffering from persistent (> 6 months) cytopenia or exhibit unexplained dysplasia without marked cytopenia. In these patients, repeated BM investigations and an extensive search for an underlying disease are usually initiated. Repeated tests in the follow up may reveal an underlying hematologic or non-hematologic disease or imminent MDS. If this is not the case, a provisional diagnosis should be established: in those with marked and persistent cytopenia (hemoglobin <10 g/dL and/or neutrophils <1,000/μL and/or platelets <100,000/μL) but no evident dysplasia (<10% of cells in major BM lineages) the diagnosis Idiopathic Cytopenia of Undetermined (Uncertain) Significance (ICUS) is established [7,57]. In those patients who have marked dysplasia (≥ 10% in at least one major lineage) with or without an MDS-related karyotype but no or only mild cytopenia, the term Idiopathic Dysplasia of Undetermined (Uncertain) Significance (IDUS) should be applied (Table 4) [57]. By definition the presence of both ICUS and IDUS is exclusive since coexistence of these conditions is diagnostic and meets criteria for MDS [57]. Some of these IDUS patients progress to frank MDS over time, whereas others may progress to a myelodysplastic/myeloproliferative neoplasm. All patients with ICUS and IDUS should have a hematologic follow-up in order to document or exclude evolution to MDS. One of the most important diagnostic investigations in patients with IDUS and ICUS is the histopathological examination of the BM. In fact, the diagnosis ICUS can only be established when the hematopathologist confirms the absence of dysplasia, and excludes all other BM disorders including aplastic anemia and hairy cell leukemia. For the same reason, the diagnosis IDUS is also dependent on the final report of the hematopathologist who has to exclude a number of differential diagnoses and can confirm multilineage dysplasia. The faculty also discussed minimal diagnostic criteria for IDUS, and concluded that the presence of dysplasia in at least two BM lineages would allow for a more proper diagnosis of IDUS than has been proposed before, where mild dysplasia in only one BM lineage might still be a questionable condition, not fulfilling the criteria of a clearly dysplastic myelopoiesis.

**Table 4:** Comparison of Criteria Defining Idiopathic Cytopenia of Unknown Significance (ICUS) and Idiopathic Dysplasia of Unknown Significance (IDUS)

Diagnosis/Condition	Defining Criteria	Additional Features
ICUS	Constant marked cytopenia* No MDS detected by criteria No dysplasia/karyotype** No MDS-Co-Criteria*** No other disease as a primary reason for cytopenia found	elderly patients EPO levels low FISH may reveal a small MDS-clone in the bone marrow
IDUS	No constant marked cytopenia* No MDS found by criteria Dysplasia and/or karyotype** No other disease as reason for Dysplasia/karyotype detected	often young patients usually detected in a routine blood test (e.g. ‘Pelger forms’ or macrocytosis)
MDS, myelodysplastic syndromes; EPO, erythropoietin; FISH, fluorescence in situ hybridization; BM, bone marrow. *constant: at least 6 months: marked: hemoglobin <10 g/dL, neutrophils <1,000/μL blood, platelets <100,000/μL; **diagnostic dysplasia: ≥10% of cells in one or more major hematopoietic lineages; karyotypes typically found in MDS; it is important to note that the diagnosis IDUS should only be established when clear signs of dysplasia in at least two hematopoietic lineages are detectable. ***if one or more co-criteria are found in suspected MDS, the condition should be termed “highly suspicious for MDS”.		

An important diagnostic approach in patients with ICUS, IDUS, and MDS, is fluorescence in situ hybridization (FISH) of BM interphase cells, especially when conventional chromosome analysis showed a normal karyotype or yielded unclear results. In several of these patients, FISH may reveal the presence of a small population of clonal cells carrying an MDS-related cytogenetic defect [57]. Sometimes, when recorded over time, the size of the clone (number of “FISH-positive” cells) may increase, BM function (i.e. the number of colony-forming progenitors) decreases, and MDS can then be diagnosed. Although IDUS may not be a rare condition, the number of well-documented cases is very low. Similar to patients with ICUS, patients with IDUS should have a hematologic follow up in order to document or exclude evolution to MDS. BM studies should be repeated when cytopenia develops or other signs for evolution to frank MDS are found. However, not all patients with ICUS or IDUS develop MDS even when recorded over many years.

## CONCLUDING REMARKS AND FUTURE PERSPECTIVES

Although parameters, assays, and score systems have improved markedly over the past two decades, appropriate diagnosis and optimal prognostication of MDS remains a challenge in clinical practice. A recommended approach is to proceed in a step wise fashion. In a first step, the diagnosis MDS should be confirmed by minimal diagnostic criteria. In a second step, the WHO classification is applied to define the disease subtype. Important prognostic markers which should always be integrated in the report include the presence and grade of BM fibrosis and the AMA-CD34. Then, the IPSS or WPSS are applied for prognostication. Finally, therapy-specific scores including the EPO-score, co-morbidity score and transplant-score are applied in order to better decide what treatment options are best indicated for the given patient. For patients with ICUS and IDUS, the general recommendation is to manage the patient in the same way as patients who have low risk MDS. All these recommendations should facilitate the management and may improve the clinical outcomes in MDS.

## References

[1] Steensma DP (2007). The spectrum of molecular aberrations in myelodysplastic syndromes: in the shadow of acute myeloid leukemia. Haematologica.

[2] Nimer SD (2008). Myelodysplastic syndromes. Blood.

[3] Orazi A, Czader MB (2009). Myelodysplastic syndromes. Am J Clin Pathol.

[4] Bennett JM, Catovsky D, Daniel MT, Flandrin G, Galton DA, Gralnick HR, Sultan C (1982). Proposals for the classification of the myelodysplastic syndromes. Br J Haematol.

[5] Brunning RD, Orazi A, Germing U, Le Beau MM, Porwit A, Baumann I, Swerdlow SH, Campo E, Harris NL, Jaffe ES, Pileri SA, Stein H, Thiele J, Vardiman JW (2008). Myelodysplastic syndromes/neoplasms. World Health Organization Classification of Tumours. Pathology & Genetics. Tumours of Haematopoietic and Lymphoid Tissues.

[6] Komrokji RS, Zhang L, Bennett JM (2010). Myelodysplastic syndromes classification and risk stratification. Hematol Oncol Clin North Am.

[7] Valent P, Horny HP, Bennett JM, Fonatsch C, Germing U, Greenberg P (2007). Definitions and standards in the diagnosis and treatment of the myelodysplastic syndromes: Consensus statements and report from a working conference. Leuk Res.

[8] Greenberg P, Cox C, LeBeau MM, Fenaux P, Morel P, Sanz G (1997). International scoring system for evaluating prognosis in myelodysplastic syndromes. Blood.

[9] Malcovati L, Germing U, Kuendgen A, Della Porta MG, Pascutto C, Invernizzi R (2007). Time-dependent prognostic scoring system for predicting survival and leukemic evolution in myelodysplastic syndromes. J Clin Oncol.

[10] Aul C, Giagounidis A, Heinsch M, Germing U, Ganser A (2004). Prognostic indicators and scoring systems for predicting outcome in patients with myelodysplastic syndromes. Rev Clin Exp Hematol.

[11] Mufti GJ, Bennett JM, Goasguen J, Bain BJ, Baumann I, Brunning R (2008). Diagnosis and classification of myelodysplastic syndrome: International Working Group on Morphology of myelodysplastic syndrome (IWGM-MDS) consensus proposals for the definition and enumeration of myeloblasts and ring sideroblasts. Haematologica.

[12] Horny HP, Sotlar K, Valent P (2007). Diagnostic value of histology and immunohistochemistry in myelodysplastic syndromes. Leuk Res.

[13] Bennett JM, Orazi A (2009). Diagnostic criteria to distinguish hypocellular acute myeloid leukemia from hypocellular myelodysplastic syndromes and aplastic anemia: recommendations for a standardized approach. Haematologica.

[14] Orazi A (2007). Histopathology in the diagnosis and classification of acute myeloid leukemia, myelodysplastic syndromes, and myelodysplastic/myeloproliferative diseases. Pathobiology.

[15] Tuzuner N, Bennett JM (1994). Reference standards for bone marrow cellularity. Leuk Res.

[16] Thiele J, Kvasnicka HM, Facchetti F, Franco V, van der Walt J, Orazi A (2005). European consensus on grading bone marrow fibrosis and assessment of cellularity. Haematologica.

[17] Lundberg LG, Hellström-Lindberg E, Kanter-Lewensohn L, Lerner R, Palmblad J (2006). Angiogenesis in relation to clinical stage, apoptosis and prognostic score in myelodysplastic syndromes. Leuk Res.

[18] Horny HP, Wehrmann M, Schlicker HU, Eichstaedt A, Clemens MR, Kaiserling E (1995). QBEND10 for the diagnosis of myelodysplastic syndromes in routinely processed bone marrow biopsy specimens. J Clin Pathol.

[19] Soligo DA, Oriani A, Annaloro C, Cortelezzi A, Calori R, Pozzoli E, Nosella D, Orazi A, Deliliers GL (1994). CD34 immunohistochemistry of bone marrow biopsies: prognostic significance in primary myelodysplastic syndromes. Am J Hematol.

[20] Bain BJ, Thompson EM (2010). Expression of CD117 by proerythroblasts. Am J Hematol.

[21] Tricot G, De Wolf-Peeters C, Vlietinck R, Verwilghen RL (1984). Bone marrow histology in myelodysplastic syndromes. II. Prognostic value of abnormal localization of immature precursors in MDS. Br J Haematol.

[22] Sperr WR, Stehberger B, Wimazal F, Baghestanian M, Schwartz LB, Kundi M (2002). Serum tryptase measurements in patients with myelodysplastic syndromes. Leuk Lymphoma.

[23] Agis H, Krauth MT, Böhm A, Mosberger I, Müllauer L, Simonitsch-Klupp I (2006). Identification of basogranulin (BB1) as a novel immunohistochemical marker of basophils in normal bone marrow and patients with myeloproliferative disorders. Am J Clin Pathol.

[24] Agis H, Krauth MT, Mosberger I, Müllauer L, Simonitsch-Klupp I, Schwartz LB (2006). Enumeration and immunohistochemical characterisation of bone marrow basophils in myeloproliferative disorders using the basophil specific monoclonal antibody 2D7. J Clin Pathol.

[25] Orazi A, Germing U (2008). The myelodysplastic/myeloproliferative neoplasms: myeloproliferative diseases with dysplastic features. Leukemia.

[26] Orazi A, Albitar M, Heerema NA, Haskins S, Neiman RS (1997). Hypoplastic myelodysplastic syndromes can be distinguished from acquired aplastic anemia by CD34 and PCNA immunostaining of bone marrow biopsy specimens. Am J Clin Pathol.

[27] Scott BL, Storer BE, Greene JE, Hackman RC, Appelbaum FR, Deeg HJ (2007). Marrow fibrosis as a risk factor for posttransplantation outcome in patients with advanced myelodysplastic syndrome or acute myeloid leukemia with multilineage dysplasia. Biol Blood Marrow Transplant.

[28] Büsche G, Teoman H, Wilczak W, Ganser A, Hecker H, Wilkens L (2008). Marrow fibrosis predicts early fatal marrow failure in patients with myelodysplastic syndromes. Leukemia.

[29] Della Porta MG, Malcovati L, Boveri E, Travaglino E, Pietra D, Pascutto C (2009). Clinical relevance of bone marrow fibrosis and CD34-positive cell clusters in primary myelodysplastic syndromes. J Clin Oncol.

[30] Olsen RJ, Dunphy CH, O'Malley DP, Rice L, Ewton AA, Chang CC (2008). The implication of identifying JAK2 (V617F) in myeloproliferative neoplasms and myelodysplastic syndromes with bone marrow fibrosis. J Hematop.

[31] Valent P, Sperr WR, Samorapoompichit P, Geissler K, Lechner K, Horny HP, Bennett JM (2001). Myelomastocytic overlap syndromes: biology, criteria, and relationship to mastocytosis. Leuk Res.

[32] Horny HP, Sotlar K, Sperr WR, Valent P (2004). Systemic mastocytosis with associated clonal haematological non-mast cell lineage diseases: a histopathological challenge. J Clin Pathol.

[33] Lim KH, Tefferi A, Lasho TL, Finke C, Patnaik M, Butterfield JH (2009). Systemic mastocytosis in 342 consecutive adults: survival studies and prognostic factors. Blood.

[34] Valent P, Horny HP, Escribano L, Longley BJ, Li CY, Schwartz LB (2001). Diagnostic criteria and classification of mastocytosis: a consensus proposal. Leuk Res.

[35] Valent P, Akin C, Escribano L, Födinger M, Hartmann K, Brockow K (2007). Standards and standardization in mastocytosis: consensus statements on diagnostics, treatment recommendations and response criteria. Eur J Clin Invest.

[36] Wang SA, Tang G, Fadare O, Hao S, Raza A, Woda BA, Hasserjian RP (2008). Erythroid-predominant myelodysplastic syndromes: enumeration of blasts from nonerythroid rather than total marrow cells provides superior risk stratification. Mod Pathol.

[37] Bennett JM, Catovsky D, Daniel MT, Flandrin G, Galton DA, Gralnick HR, Sultan C (1985). Proposed revised criteria for the classification of acute myeloid leukemia. A report of the French-American-British Cooperative Group. Ann Intern Med.

[38] Goldberg SL, Noel P, Klumpp TR, Dewald GW (1998). The erythroid leukemias: a comparative study of erythroleukemia (FAB M6) and Di Guglielmo disease. Am J Clin Oncol.

[39] Hasserjian RP, Zuo Z, Garcia C, Tang G, Kasyan A, Luthra R (2010). Acute erythroid leukemia: a reassessment using criteria refined in the 2008 WHO classification. Blood.

[40] Bennett JM, Catovsky D, Daniel MT, Flandrin G, Galton DA, Gralnick H, Sultan C, Cox C (1994). The chronic myeloid leukaemias: guidelines for distinguishing chronic granulocytic, atypical chronic myeloid, and chronic myelomonocytic leukaemia. Proposals by the French-American-British Cooperative Leukaemia Group. Br J Haematol.

[41] Germing U, Strupp C, Knipp S, Kuendgen A, Giagounidis A, Hildebrandt B (2007). Chronic myelomonocytic leukemia in the light of the WHO proposals. Haematologica.

[42] Orazi A, Chiu R, O'Malley DP, Czader M, Allen SL, An C, Vance GH (2006). Chronic myelomonocytic leukemia: The role of bone marrow biopsy immunohistology. Mod Pathol.

[43] Qubaja M, Marmey B, Le Tourneau A, Haiat S, Cazals-Hatem D, Fabiani B, Diebold J, Marie JP, Audouin J, Geissmann F, Molina TJ (2009). The detection of CD14 and CD16 in paraffin-embedded bone marrow biopsies is useful for the diagnosis of chronic myelomonocytic leukemia. Virchows Arch.

[44] Bacher U, Haferlach T, Kern W, Haferlach C, Schnittger S (2007). A comparative study of molecular mutations in 381 patients with myelodysplastic syndrome and in 4130 patients with acute myeloid leukemia. Haematologica.

[45] Kohlmann A, Grossmann V, Klein HU, Schindela S, Weiss T, Kazak B (2010). Next-generation sequencing technology reveals a characteristic pattern of molecular mutations in 72.8% of chronic myelomonocytic leukemia by detecting frequent alterations in TET2, CBL, RAS, and RUNX1. J Clin Oncol.

[46] Valent P, Samorapoompichit P, Sperr WR, Horny HP, Lechner K (2002). Myelomastocytic leukemia: myeloid neoplasm characterized by partial differentiation of mast cell-lineage cells. Hematol J.

[47] Sperr WR, Drach J, Hauswirth AW, Ackermann J, Mitterbauer M, Mitterbauer G (2005). Myelomastocytic leukemia: evidence for the origin of mast cells from the leukemic clone and eradication by allogeneic stem cell transplantation. Clin Cancer Res.

[48] Arredondo AR, Gotlib J, Shier L, Medeiros B, Wong K, Cherry A (2010). Myelomastocytic leukemia versus mast cell leukemia versus systemic mastocytosis associated with acute myeloid leukemia: a diagnostic challenge. Am J Hematol.

[49] Tefferi A, Patnaik MM, Pardanani A (2006). Eosinophilia: secondary, clonal and idiopathic. Br J Haematol.

[50] Gotlib J, Cross NC, Gilliland DG (2006). Eosinophilic disorders: molecular pathogenesis, new classification, and modern therapy. Best Pract Res Clin Haematol.

[51] Valent P (2009). Pathogenesis, classification, and therapy of eosinophilia and eosinophil disorders. Blood Rev.

[52] Cools J, DeAngelo DJ, Gotlib J, Stover EH, Legare RD, Cortes J (2003). A tyrosine kinase created by fusion of the PDGFRA and FIP1L1 genes as a therapeutic target of imatinib in idiopathic hypereosinophilic syndrome. N Engl J Med.

[53] Gotlib J, Cools J (2008). Five years since the discovery of FIP1L1-PDGFRA: what we have learned about the fusion and other molecularly defined eosinophilias. Leukemia.

[54] Samorapoompichit P, Kiener HP, Schernthaner GH, Jordan JH, Agis H, Wimazal F (2001). Detection of tryptase in cytoplasmic granules of basophils in patients with chronic myeloid leukemia and other myeloid neoplasms. Blood.

[55] van de Loosdrecht AA, Alhan C, Béné MC, Della Porta MG, Dräger AM, Feuillard J (2009). Standardization of flow cytometry in myelodysplastic syndromes: report from the first European LeukemiaNet working conference on flow cytometry in myelodysplastic syndromes. Haematologica.

[56] Ecker RC, de Martin R, Steiner GE, Schmid JA (2004). Application of spectral imaging microscopy in cytomics and fluorescence resonance energy transfer (FRET) analysis. Cytometry A.

[57] Valent P, Horny HP (2009). Minimal diagnostic criteria for myelodysplastic syndromes and separation from ICUS and IDUS: update and open questions. Eur J Clin Invest.

